# Forensic Evaluation of Multiple Gunshot Injuries: A Case Report

**DOI:** 10.7759/cureus.85439

**Published:** 2025-06-05

**Authors:** Treasa James, Akhilesh Pathak

**Affiliations:** 1 Forensic Medicine, All India Institute of Medical Sciences, Bathinda, IND

**Keywords:** ballistics, firearm, forensic, lead, poisoning, shotgun

## Abstract

Firearm injuries, particularly those caused by shotguns, present complex clinical and forensic challenges due to the variable nature of wound patterns and retained projectiles. Shotguns inflict varying injury patterns based on range and ammunition type, while high-velocity bullets cause extensive cavitation injuries, especially in dense or fluid-filled organs. Understanding firearm mechanisms, wound ballistics, and associated forensic evidence like gunpowder residue and blood patterns is essential for accurate forensic investigation, trauma management, and preventing long-term complications from retained projectiles.

This case report describes the evaluation and conservative management of a 26-year-old male patient with multiple gunshot wounds sustained at long range. Clinical assessment revealed multiple entry wounds on the left thigh, abdomen, and left middle finger, without evidence of close-range indicators such as tattooing or burning. Radiological imaging confirmed the presence of multiple retained shotgun pellets lodged in soft tissue, with no associated fractures or critical organ damage. The patient remained hemodynamically stable and showed no signs of infection, lead toxicity, or neurological deficits. He was managed conservatively and discharged on the third day. Follow-up at six months revealed no complications, and the patient resumed normal activities. Comparative literature review highlights similar cases where retained bullet fragments remained asymptomatic, emphasizing that surgical removal is not always warranted unless complications arise.

This case illustrates the significance of ballistic analysis, radiographic evaluation, and careful forensic wound assessment in gunshot injuries. It underscores the importance of individualized patient management, as retained pellets may not always lead to adverse outcomes. Furthermore, it contributes to the forensic understanding of long-range shotgun wounds and reinforces the need for thorough documentation and follow-up in such cases. Overall, this report serves as a critical intersection of forensic medicine, clinical management, and ballistics, emphasizing the importance of a multidisciplinary approach in handling firearm-related trauma.

## Introduction

There are particular forensic and clinical difficulties with firearm-related injuries, especially those brought on by shotguns. The mechanics of projectiles, especially bullets, and their behavior both in flight and at impact are studied in ballistics. It is divided into three categories: terminal ballistics (the impact of the bullet on the target), external ballistics (the trajectory and flight path of the bullet), and internal ballistics (the propulsion of the bullet within the firearm). Internal ballistics includes elements that affect bullet acceleration, such as chamber and barrel rifling, and propellant ignition. The study of external ballistics looks at how air resistance, friction, and gravity impact a bullet's path. Terminal ballistics examines the impact of the bullet while taking tissue damage, weapon type, and bullet characteristics into account. Forensic investigations, weapon design, and shooting accuracy all depend on an understanding of ballistics [1].

Shotguns are smoothbore weapons that can fire a variety of rounds, such as slugs, buckshot, and birdshot. They come in a variety of action types, including breach load, pump-action, lever-action, and semiautomatic, and unlike rifles, they do not have rifling inside the barrel. A shotgun's calibre is measured in gauge; a larger bore diameter is indicated by a lower gauge number [1,2].

The type of ammunition and firing range determine the impact of shotgun wounds. Pellets act as a single mass at close range (less than 3 yards), destroying large amounts of tissue. While buckshot can still penetrate deeply, birdshot usually causes superficial injuries at longer ranges (beyond 7 yards), while they disperse at intermediate range (3-7 yards), resulting in multiple penetrating injuries. Buckshot rounds have a high-energy impact, so injuries frequently require surgical treatment [2,3].

The main ways that gunshot wounds damage tissue are by laceration, crushing, and cavitation. The bullet's shear forces as it passes through the body cause laceration and crushing injuries. Bullets typically travel in a straight line, but high-energy projectiles can lose stability and yaw, pitch, or tumble, which increases tissue damage. Unless they hit bone, which could result in fragmentation, handgun bullets normally travel in a linear trajectory. By more efficiently transferring kinetic energy, certain bullets, like hollow points, are made to expand upon impact, increasing tissue damage [3,4].

When high-velocity bullets cause cavitation injuries, the pressure wave that displaces tissues creates a temporary cavity as well as a permanent cavity (the direct wound track). Tissue density and elasticity determine how much cavitation occurs; solid, inelastic organs like the liver, spleen, kidneys, and brain are more likely to split or shatter. On the other hand, the intestines, muscles, and skin absorb energy more effectively and are less prone to secondary cavitation. Due to propellant gases entering the wound, close-range gunshot wounds may result in further tissue expansion. Extensive cavitation injuries from high-velocity gunshot wounds frequently cause severe tissue destruction, especially in abdominal wounds where the force of impact may cause evisceration [5,6].

The design, calibre, and firing mechanisms of modern firearms vary, and they are categorized according to elements like bore diameter and action type (e.g., revolvers, semiautomatic, automatic, bolt-action). In general, firearms can be classified as either high-velocity (such as rifles) or low-velocity (such as handguns).

Single-shot pistols, revolvers, and semiautomatic pistols are examples of handguns, which are intended for one-handed use and self-defense. Single-shot pistols need to be reloaded after every shot because they can only hold one round. Revolvers are available in single-action (which requires manual hammer cocking) and double-action (where pulling the trigger cocks and fires the gun in one motion) varieties. They feature a rotating cylinder that enables multiple rounds to be fired without reloading. With a magazine and a fixed firing chamber, semiautomatic pistols allow for quick successions of shots while the recoil automatically chambers the subsequent round. These weapons, which are frequently used for self-defense, have magazines that hold 7-17 rounds; in certain states, high-capacity magazines that hold more than 10 rounds are prohibited [1,2].

Long arms, such as shotguns and rifles, need both hands for stability because of their longer barrels. Internal rifling or spiral grooves in rifles rotate the bullet for range and accuracy. They are available in bolt-action, automatic, semiautomatic, and single-shot types. Semiautomatic rifles chamber the subsequent round using recoil, whereas bolt-action rifles need to be manually reloaded after every shot. Frequently used in military environments, assault rifles use intermediate-sized cartridges and can fire selectively (automatic, semiautomatic, or burst mode). In contrast to rifles, shotguns have a smooth bore and can fire a variety of projectiles, including slugs, buckshot, and birdshot. Their calibre is expressed in gauge, where a larger bore diameter is indicated by a lower gauge number. The range of fire determines the extent of shotgun injuries; long-range shots spread pellets over a wider area, reducing penetration, while close-range wounds cause significant damage because the pellets behave like a single mass.

The type of tissue affected, the distance to the target, and the muzzle kinetic energy all affect how severely a firearm damages tissue. The kinetic energy of a bullet is transferred to the tissue when it strikes, causing more damage to denser, less elastic organs (such as the liver, spleen, and brain) than to more elastic tissues (such as the skin, lungs, and muscle). Because they cannot compress and may burst upon impact, fluid-filled structures like the bladder, heart, great vessels, and bowel are especially susceptible. Bone fractures are frequent, and secondary fragments from bullet impacts frequently serve as extra projectiles, further injuring nearby tissues. Forensic investigations, trauma treatment, and law enforcement evaluations all depend on an understanding of firearm mechanisms and their effects on the human body [1,3,6].

The shooting distance and the position of the entrance and exit wounds are important indicators of how a firearm was used. Reconstructing a crime scene requires the use of additional forensic evidence, such as gunpowder residue and bloodstain patterns (such as backspatter). The distribution of pellets, tissue properties, and gas pressure in close-range shots all affect how severe a shotgun wound is [7]. The characteristics of the target tissue (density, elasticity, and viscosity) and the bullet (calibre, mass, velocity, shape, and material) all affect how much soft tissue damage occurs. It can occasionally be challenging to tell the difference between entrance and exit wounds from close-range gunshot wounds because they often result in more serious damage than long-range shots [8].

Toxicology and infection result from a foreign object in the body [9,10]. By encasing themselves in a granulation tissue reaction, certain foreign objects manage to stay isolated and present minimal risk. Retained shotgun pellets may cause delayed complications like infection, toxicity, or migration to vital organs, but they can also occasionally go years without causing any symptoms, according to research [11,12].

This case study highlights the significance of forensic wound assessment, ballistics interpretation, and proper medical management by presenting the forensic and clinical evaluation of a 26-year-old man who had multiple gunshot wounds. This case report details a gunshot wound in which several pellets stayed embedded in different body parts without resulting in severe hematoma formation or active bleeding. Notably, there were no symptoms or indications of fracture despite the retained pellets, and their removal did not require surgery. Surgical intervention in firearm injuries is generally deferred in hemodynamically stable patients without acute complications. Given the potential risks of pellet migration and surgical morbidity, this case highlights a unique presentation where embedded projectiles caused no significant side effects or need for surgical removal.

## Case presentation

A 26-year-old Indian man, well-built and measuring 176 cm in height and weighing 75 kg, was brought to the emergency department with an alleged gunshot injury. The circumstances surrounding the shooting were unclear, necessitating a comprehensive forensic and medical evaluation to determine the severity of the wounds, the likely firearm range, and the potential for delayed complications.

Upon arrival at the emergency department, the patient was conscious and hemodynamically stable, with no immediate signs of life-threatening distress. A thorough general examination was conducted, revealing that bilateral air entry was present, heart sounds (S1 and S2) were normal, the abdomen was soft and non-tender, and there were no neurological deficits.

**Figure 1:**
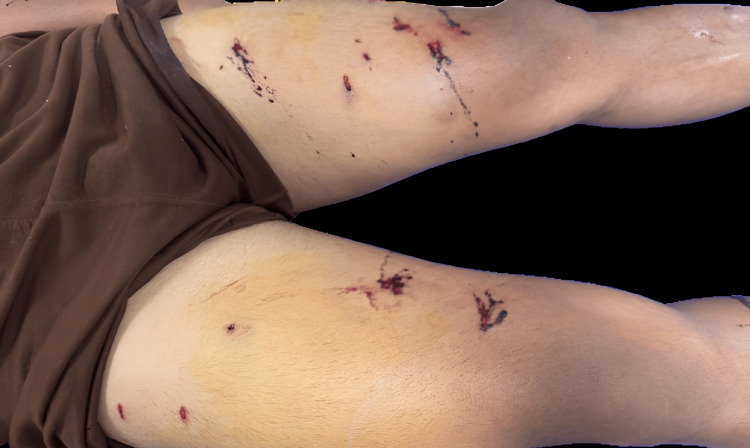
Multiple gunshot entry wounds on the left and right thighs

**Figure 2:**
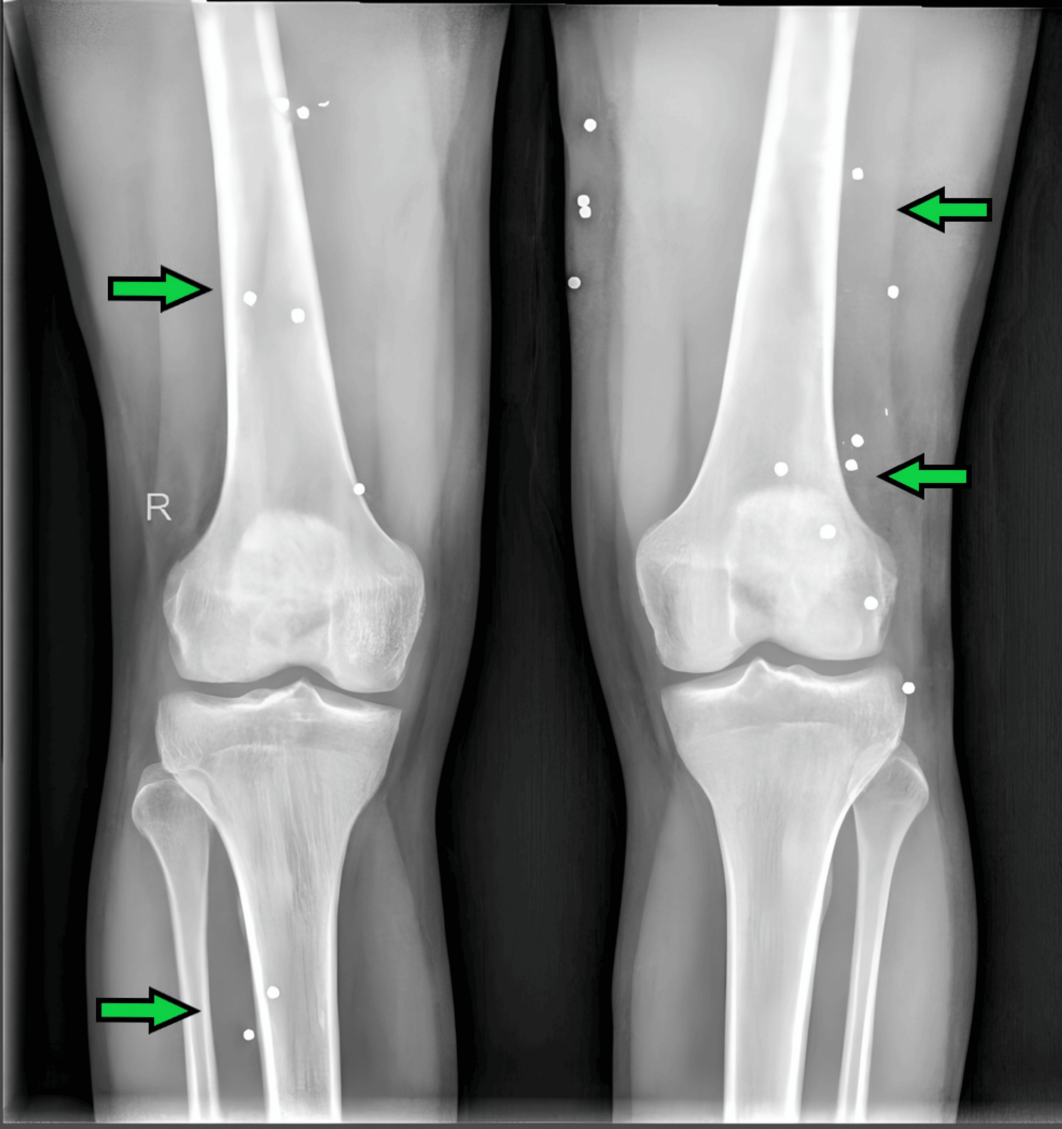
X-ray AP view of the bilateral knees with the distal thigh and upper leg showing multiple well-defined radiodense foreign bodies, which are reminiscent of tiny pellets AP: anteroposterior

On examination, the patient sustained multiple gunshot wounds on the left and right thigh, abdomen, and left middle finger, characterized by round entry wounds measuring 6-8 mm with a 0.2 cm contusion collar. The wounds exhibited fresh clotted blood, with slight trickling, but notably, there were no signs of close-range firing, such as burning, singeing, blackening, or tattooing. The left thigh had six muscle-deep wounds extending from the upper thigh to the knee, as seen in Figure 1, while the abdomen had a single cavity-deep wound located 12 cm above the anterior superior iliac spine (ASIS) and 11 cm left lateral to the midline. The left middle finger sustained three muscle-deep entry wounds. Examination of the victim's clothing revealed corresponding defects with dried blood, but no visible gunpowder, soot, or burn marks, suggesting the shots were fired from a distance rather than close range. The absence of fractures or significant hemorrhage indicated that no surgical intervention was required. The pattern of multiple entry wounds in different body regions suggests projectile dispersion, possibly from a shotgun or multiple successive shots. The forensic findings were crucial in reconstructing the shooting scenario, determining the firearm type, and assessing the shooting distance. X-ray imaging of the affected regions revealed multiple retained shotgun pellets scattered across soft tissue. No significant bony involvement or organ damage was noted. Figure 2 shows the X-ray of the bilateral knees with the distal thigh and upper leg with multiple well-defined radiodense foreign bodies, which are reminiscent of tiny pellets.

**Figure 3:**
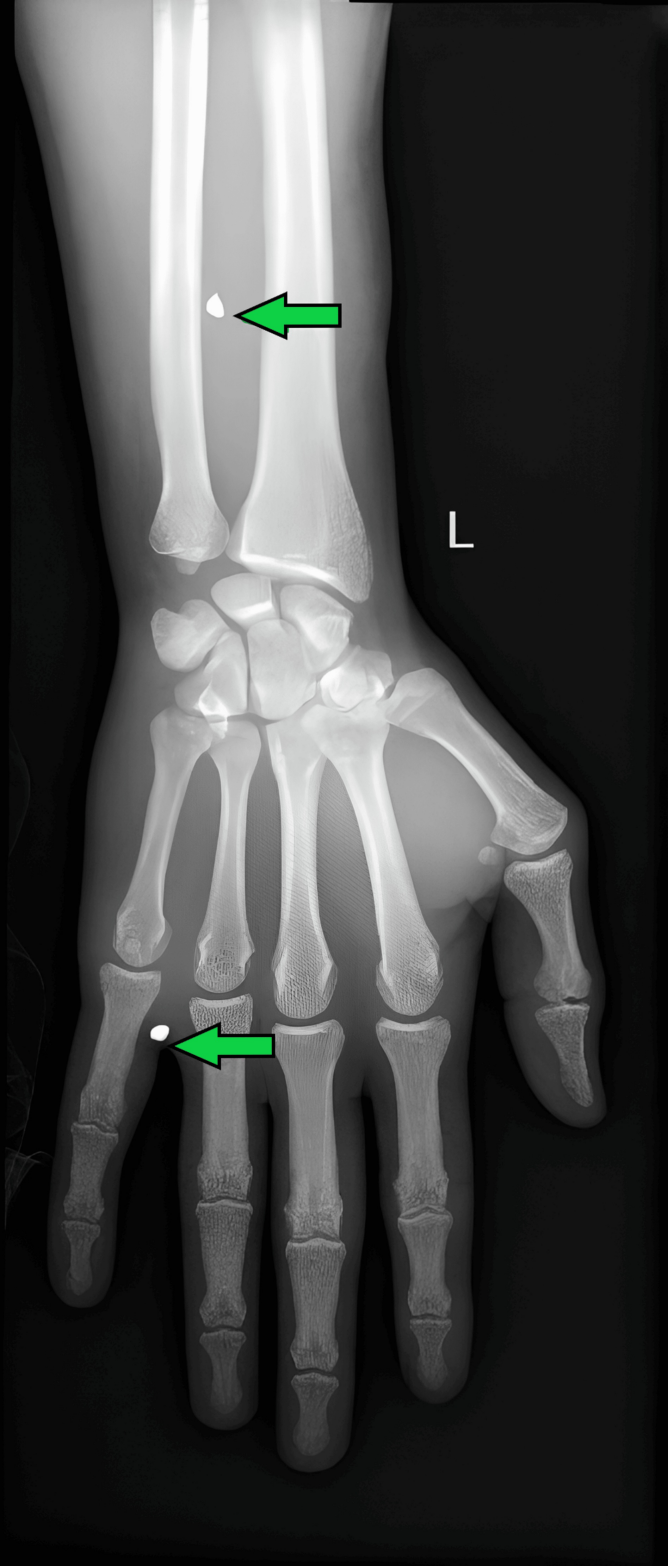
X-ray AP view of the left wrist showing tiny pellets seen in the lower one-third of the left forearm and in between the fourth and fifth proximal phalanges AP: anteroposterior

Figure 3 shows the X-ray of the left wrist, indicating tiny pellets seen in the lower one-third of the left forearm and in between the fourth and fifth proximal phalanges.

**Figure 4:**
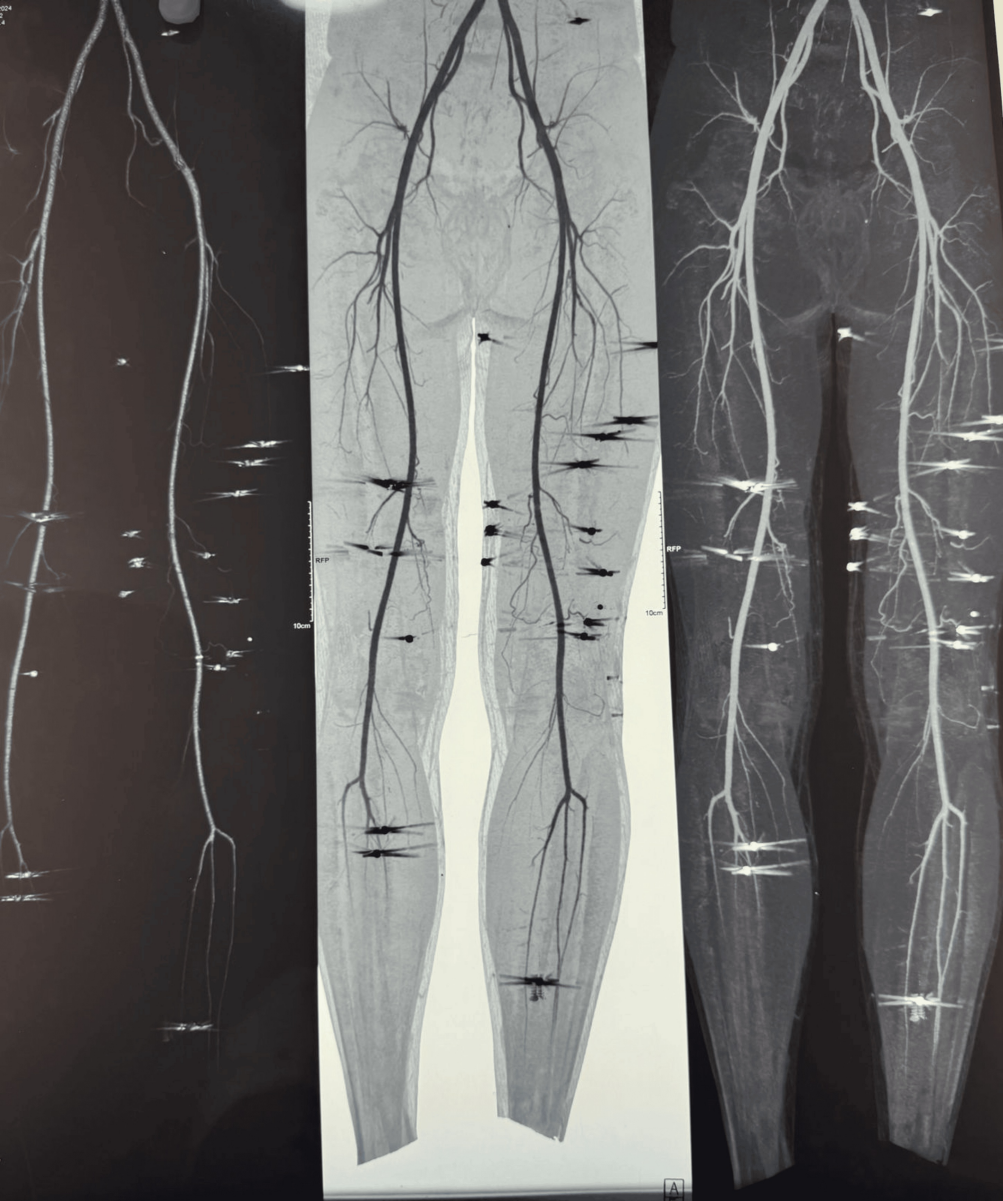
CT angiography of the bilateral lower limb arteries showing multiple tiny pellets giving streak artifacts seen in the subcutaneous and intramuscular planes of the left and right thigh and leg regions with no associated vascular injury

Figure 4 shows the CT angiography of the bilateral lower limb arteries showing multiple tiny pellets giving streak artifacts seen in the subcutaneous and intramuscular planes of the left and right thigh and leg regions with no associated vascular injury.

The radiological findings confirmed that the pellets were lodged in non-critical areas, supporting the hypothesis of a long-range shotgun injury. The pattern of pellet dispersion further indicated that the injury was inflicted from a distance, where individual pellets had started to spread widely, rather than acting as a single mass. The patient was managed conservatively and discharged on the third day. 

At the six-month follow-up, the patient remained asymptomatic, with no signs of infection or lead poisoning. Repeated X-rays showed no migration or complications related to the retained pellets, and the patient was able to carry out his daily activities without any restrictions. Blood lead levels were assessed during follow-up and found to be within normal limits, supporting the absence of lead toxicity despite the retention of multiple pellets in soft tissues. Additionally, a metal detector card was provided to assist in future medical evaluations and screening for retained pellets.

**Figure 5:**
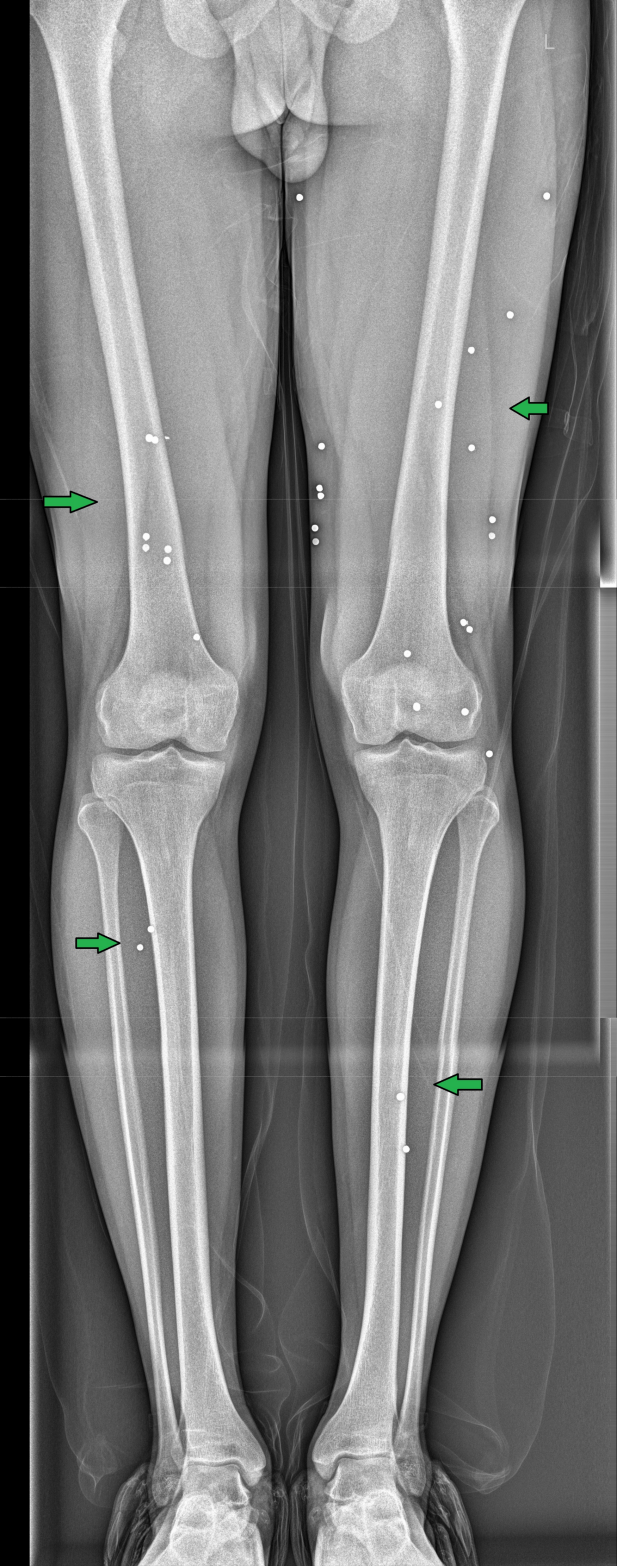
Full-length X-ray of both legs with the distal thigh and upper leg showing multiple well-defined radiodense foreign bodies which are reminiscent of tiny pellets. In comparison with the previous X-ray, there are no significant interval changes

Figure 5 shows the full-length X-ray of both legs, with the distal thigh and upper leg showing multiple well-defined radiodense foreign bodies, which are reminiscent of tiny pellets. In comparison with the previous X-ray, there are no significant interval changes.

**Figure 6:**
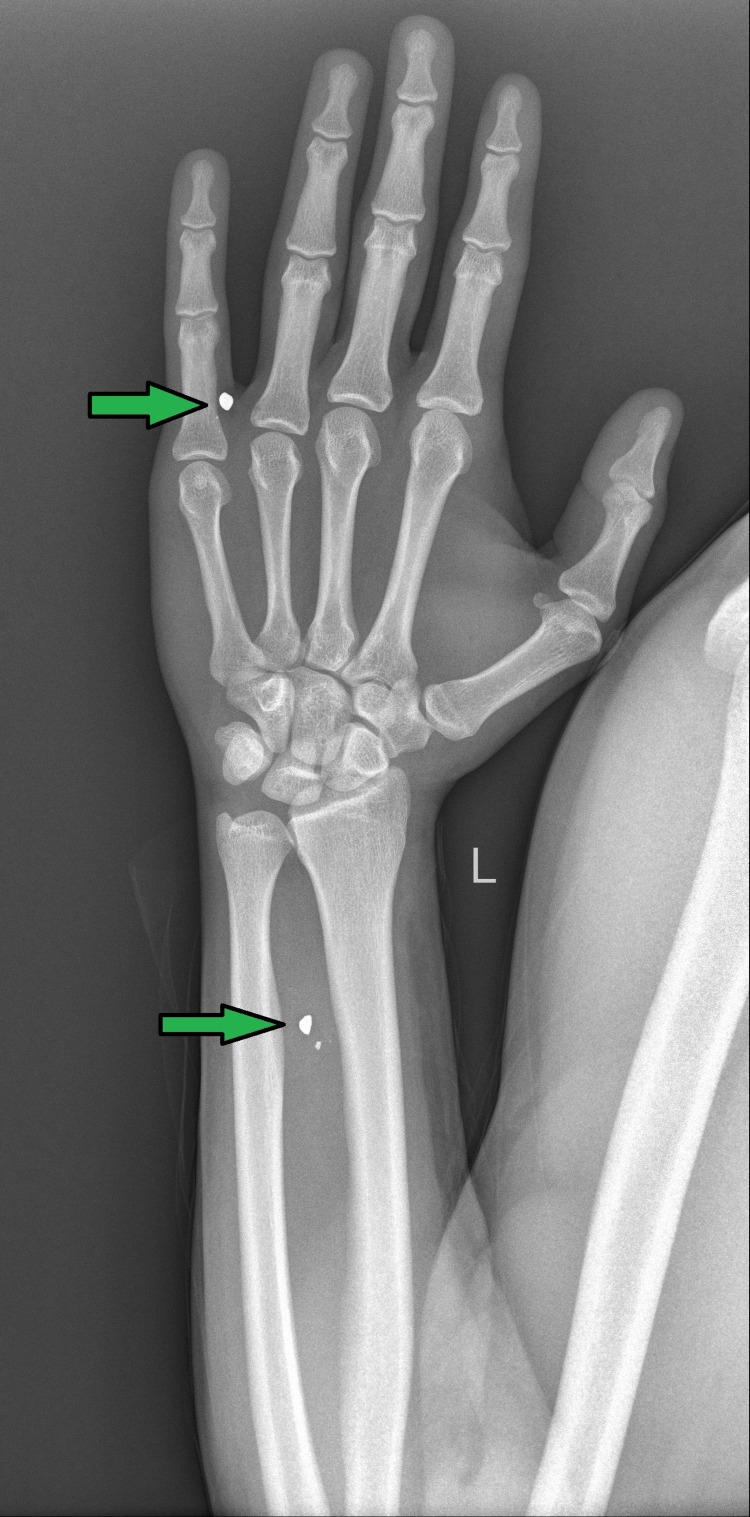
X-ray AP view of the left wrist showing tiny pellets seen in the lower one-third of the left forearm and in between the fourth and fifth proximal phalanges. In comparison with the previous X-ray, there are no significant interval changes AP: anteroposterior

Figure 6 shows the X-ray of the left wrist with tiny pellets seen in the lower one-third of the left forearm and in between the fourth and fifth proximal phalanges. In comparison with the previous X-ray, there are no significant interval changes.

## Discussion

Depending on the range, type of ammunition, and anatomical involvement, shotgun injuries can vary greatly. While the spread pattern at long range results in numerous dispersed wounds with limited penetration, at close range, pellets behave as a single mass, causing severe penetrating injuries. Forensic ballistics analysis was essential in reconstructing the incident because the absence of close-range indicators in this instance suggested a long-range shotgun discharge.

Fernandes and Fernandes [3] in their article highlighted the possible long-term effects of retained foreign bodies in the oral and maxillofacial region by presenting a unique case report of a 50-year-old man who had multiple lead bullets lodged in his mandible for more than 12 years after a gunshot injury. Even though there was a high concentration of lead bullets, the patient showed no discernible symptoms of infection or lead toxicity. Histopathological analysis demonstrated persistent non-specific inflammation and hyperplasia, while radiographic examinations showed sporadic radiopacities in the mandible and surrounding soft tissue. Blood lead levels were slightly elevated but not toxic, suggesting that the bullets were encapsulated by fibrotic tissue, preventing significant lead dissolution into the bloodstream. The article emphasizes the importance of considering the removal of foreign bodies only when there is a serious health risk, such as infection or toxicity, and advises the periodic monitoring of blood lead levels, especially in older patients, due to the potential for lead mobilization during metabolic changes. The case underscores the need for careful management of retained lead bullets, including dietary recommendations, to reduce lead absorption and the use of chelating agents if toxicity arises. This report contributes to the literature by illustrating that foreign bodies can remain clinically silent for extended periods and their removal should be approached cautiously to avoid complications such as pathologic fractures. Our case differs from the one presented by Fernandes and Fernandes [3] in several key aspects. While their case involved a single gunshot injury with multiple lead bullets lodged in the mandible for over 12 years, our case involves multiple gunshot wounds affecting different body regions, including the left thigh, abdomen, and left middle finger, with pellets retained in various tissues. Additionally, our patient did not require surgical intervention, as the retained pellets did not cause fractures or systemic complications, whereas in the Fernandes and Fernandes case, surgical removal was necessary for some bullets due to prosthetic rehabilitation needs.

de Madureira et al. [4] in their article presented a case report of a 23-year-old man who developed clinical lead intoxication several years after sustaining a gunshot wound, highlighting the potential risks of retained lead bullet fragments in the body, particularly when lodged in large joints such as the hip. The patient presented with recurrent abdominal pain, weakness, vomiting, and diarrhea, which were initially misdiagnosed but later attributed to lead poisoning after radiographic evidence revealed a bullet fragment in the left hip joint. Blood and urine tests confirmed elevated lead levels, prompting chelation therapy with calcium versenate, which provided temporary relief. However, due to the recurrence of symptoms caused by chronic lead mobilization from the joint, a definitive solution was achieved through hip arthroplasty, which removed the source of lead and resolved the symptoms. The case underscored the importance of considering lead poisoning in patients with retained bullet fragments, especially in synovial joints, where lead can dissolve into synovial fluid and cause systemic toxicity. The article emphasized the need for close follow-up and early surgical intervention in such cases to prevent long-term complications, as well as the role of chelation therapy as a palliative measure prior to definitive treatment. The study by de Madureira et al. [4], which focused on lead intoxication due to retained bullet fragments in a large joint (hip), causing systemic toxicity over time, differs significantly from our case which involves multiple gunshot wounds with retained pellets in soft tissues but no clinical signs of lead poisoning, fractures, or complications. While their patient developed abdominal pain, vomiting, and weakness, requiring chelation therapy and surgical removal via hip arthroplasty, our patient remained clinically stable, highlighting that retained pellet fragments do not always cause systemic toxicity and surgical removal should be considered only if complications arise.

From a forensic perspective, retained shotgun pellets necessitate careful documentation and follow-up. While some embedded pellets remain clinically insignificant, others may lead to delayed complications, such as infection due to foreign body retention, lead toxicity, or pellet migration, which can cause secondary organ damage. Previous studies have reported cases where gunshot fragments remained inside the body for years without causing harm, only requiring removal if they became symptomatic. In this particular case, given the absence of systemic toxicity, infection, or functional impairment, the patient was managed conservatively without the need for surgical removal.

The article by Baydilli et al. [5] presented a rare case of a 26-year-old man who sustained a shotgun injury to the penis, with a pellet remaining in the left corpus cavernosum without causing any symptoms of penile fracture or significant complications. The patient was treated conservatively without surgical intervention, as there was no active bleeding, hematoma, or rupture of the tunica albuginea. This case is notable for being the first reported instance of a pellet retained in the corpus cavernosum following a gunshot injury that did not require surgical removal. This case highlighted that conservative management may be appropriate in select cases where the injury is superficial and there is no evidence of structural damage. The article also discussed the potential for pellet migration over time, a phenomenon documented in other cases where foreign bodies have moved within the body, sometimes requiring surgical removal. In contrast to the case reported by Baydilli et al. [5], our patient had pellets retained in the abdomen and extremities; however, imaging and clinical follow-up showed no signs of intra-abdominal migration, and there were no associated complications, thereby supporting conservative management

Wound analysis plays a crucial role in estimating the range and type of firearm used, as the size, depth, and distribution of the wounds provide valuable insights. Clothing examination helps forensic experts match fabric defects with wounds, aiding in the reconstruction of ballistic trajectories. Radiological assessment further aids in understanding the extent of injury and the location of retained pellets, guiding medical and forensic decisions.

## Conclusions

This case underscores the complexity of shotgun injuries and highlights the critical role of forensic medicine in their comprehensive evaluation. Despite multiple gunshot wounds, the patient remained asymptomatic six months post-injury, with no evidence of lead toxicity or infection. This reinforces that not all retained gunshot fragments necessitate immediate surgical removal; in selected cases, conservative management with vigilant monitoring can be both safe and effective. Given the patient's stable clinical condition, the deep soft tissue location of the retained pellets, and the absence of complications, surgical intervention was deferred. Blood lead levels remained within normal limits at six-month follow-up, and the patient continues under close clinical surveillance with instructions to promptly report any new or concerning symptoms.

From a forensic standpoint, systematic wound analysis, ballistic interpretation, and radiological assessment are essential for accurate injury evaluation. Proper forensic documentation ensures that such cases are handled with scientific precision, aiding both medical treatment and legal proceedings. This case serves as a valuable example of the intersection of forensic pathology, ballistics, and clinical management in firearm-related injuries.
